# High-Order Harmonics Generation in MoS_2_ Transition Metal Dichalcogenides: Effect of Nickel and Carbon Nanotube Dopants

**DOI:** 10.3390/ijms24076540

**Published:** 2023-03-31

**Authors:** Mottamchetty Venkatesh, Vyacheslav V. Kim, Ganjaboy S. Boltaev, Srinivasa Rao Konda, Peter Svedlindh, Wei Li, Rashid A. Ganeev

**Affiliations:** 1 GPL Photonics Laboratory, State Key Laboratory of Applied Optics, Changchun Institute of Optics, Fine Mechanics and Physics, Chinese Academy of Sciences, Changchun 130033, China; 2 Department of Materials Science and Engineering, Uppsala University, P.O. Box 35, SE-75103 Uppsala, Sweden; 3 Laboratory of Nonlinear Optics, University of Latvia, Jelgavas 3, LV-1004 Riga, Latvia; 4 Institute of Fundamental and Applied Research, TIIAME National Research University, Kori Niyoziy 39, Tashkent 100000, Uzbekistan; 5 Faculty of Physics and Matematics, Chirchik State Pedagogical University, 104 Amir Temur, Chirchik 111700, Uzbekistan; 6 Department of Physics, Voronezh State University, 394006 Voronezh, Russia

**Keywords:** molecular plasma, high-order harmonics generation, optimization of plasma formation, resonance enhancement of harmonic

## Abstract

The transition metal dichalcogenides have instigated a lot of interest as harmonic generators due to their exceptional nonlinear optical properties. Here, the molybdenum disulfide (MoS_2_) molecular structures with dopants being in a plasma state are used to demonstrate the generation of intense high-order harmonics. The MoS_2_ nanoflakes and nickel-doped MoS_2_ nanoflakes produced stronger harmonics with higher cut-offs compared with Mo bulk and MoS_2_ bulk. Conversely, the MoS_2_ with nickel nanoparticles and carbon nanotubes (MoS_2_-NiCNT) produced weaker coherent XUV emissions than other materials, which is attributed to the influence of phase mismatch. The influence of heating and driving pulse intensities on the harmonic yield and cut-off energies are investigated in MoS_2_ molecular structures. The enhanced coherent extreme ultraviolet emission at ~32 nm (38 eV) due to the 4p-4d resonant transitions is obtained from all aforementioned molecular structures, except for MoS_2_-NiCNT.

## 1. Introduction

Transition metal dichalcogenides (TMDs), such as molybdenum disulfide (MoS_2_), are hexagonal semiconductors with transition metal atoms held together by a weak Wander Waals force between two layers of chalcogen atoms. 2D-TMDs are a developing class of materials with properties that make them particularly appealing for fundamental research and search of novel physical phenomena [1,2,3,4,5]. These materials are very compact, with some layers on a 2D atomic scale, and have a direct bandgap. As a result, they can provide a solid interface with the incident photon and have advantageous properties like broadband absorption, transparency, and high carrier mobility. The interest in 2D TMDs, particularly MoS_2_ monolayers, resulted in a variety of applications [6,7,8,9,10,11]. The bandgap of TMDs depends on the number of layers and the type of dopants. Studying the modified morphological, linear, and nonlinear optical properties of 2D TMDs is a significant research topic as they can be utilized in evolving next-generation high-performance optical devices.

Additionally, TMDs like molybdenum disulfide (MoS_2_) have received greater attention because of their large direct bandgap in few-layered material, leading to an increase in the nonlinear optical properties as compared to the bulk material [12,13]. MoS_2_ has exceptional characteristics like tunable bandgap, strong photoluminescence, carrier dynamics at ultrashort time scale, saturable absorption, strong spin-orbit coupling, etc. [3,13]. These outstanding properties of MoS_2_ created a potential to develop next-generation valleytronic, spintronic, optoelectronic, and electronic devices such as transistors, flexible sensors, optical limiters, mode-lockers, and Q-switches for ultrafast lasers, energy harvesting devices, etc. TMD/organic molecule heterostructures showed enhancement of photoluminescence (PL) intensity and charge carrier mobility leading to potential candidates for next-generation optoelectronic devices. Habib et al. performed density functional theory on TMD/organic molecules to study their interfacial and electronic properties [6]. Their study reveals modulation of electronic properties i.e., reduction of work function associated with charge transfer between layers of heterostructures has applications in LEDs. A study by Kumar et al. revealed that MoS_2_ nanosheets could be used as gas sensors and catalysts by creating sulfur monovacancies [7]. 

MoS_2_ is a molecular semiconducting material, which generates intense PL and second-order harmonic using the dopants [14,15]. Stavrou et al. reported control of the crystalline phase of MoS_2_ and WS_2_, which provides an efficient strategy for 2D nanostructures with tailored nonlinear optical properties for specific optoelectronic and photonic applications [16]. These materials have a variety of nonlinear optical properties that can be easily tailored by changing the chalcogen atom. Moreover, resonant harmonic at the wavelength *λ* = 32 nm is generated from plasmas of molybdenum species [17,18]. 

The conversion efficiency of high-order harmonics is a key issue when considering the practical applications of such extreme ultraviolet (XUV) sources. Hence, to increase the yield and cut-off energy of harmonics, different schemes like resonance-induced enhancement, quasi-phase matching of harmonics, application of multi-particle species, optimization of laser parameters, use of polarization structured beams, multicolor excitation, etc. have been introduced [19,20,21,22,23,24,25,26,27,28]. 

Recently, the role of dopants on high-order harmonics generation (HHG) from plasmas of ZnO molecular structures and CsPbBr_3_ 2D nanocrystals are investigated [29,30,31,32]. The dopant percentage affected the nonlinear optical properties of those structures, which resulted in enhancing and diminishing the yield of high-order harmonics. Moreover, the intense coherent XUV emission was generated from molecular semiconductors by doping with chromium and changing the dopants [33,34]. Similarly, one can expect that, in the case of MoS_2_ samples, the dopants enhance the low- and high-order nonlinear optical properties of these structures. Moreover, there are no studies on the influence of dopants on high-order harmonics and resonant harmonic (at 32 nm) emitted from molecular structures like MoS_2_ TMDs.

In this work, the MoS_2_ two-dimensional molecular materials having various dopants like nickel nanoparticles (NPs) and carbon nanotubes (CNT) are used as the media for high-order harmonics generation to produce intense extreme ultraviolet pulses along with the characterization of material properties. We study the effect of ions, NPs, and CNT on the harmonic cut-off and the yield of single resonant harmonic generated from MoS_2_ molecular plasmas. The cut-off photon energies and harmonic yield efficiencies of materials are ascertained by varying the intensities of driving (DP) and heating pulses (HP). The role of nickel NPs in enhancing the nonlinear optical properties of MoS_2_ and high-order harmonic yield is revealed by carrying out the Z-scan studies. The present work provides a path towards the recognition of TMDs as the efficient XUV harmonic source, which can find applications in the areas of attosecond science, lithography, spectroscopy, and imaging (i.e., with nanoscale resolutions), etc.

## 2. Results

### 2.1. HHG and Z-Scan Measurements Using Mo-Contained Molecular Plasmas and Suspensions

The high-order harmonic spectroscopy of molecular and atomic semiconductors uncovers the influence of resonance phenomenon at particular harmonic order and the specimen components’ effect on harmonic yield. We selected the molecular species (MoS_2_ semiconducting TMDs) to reveal the effect of any hidden resonance process on the yield of harmonic orders. The morphology, optical, and electrochemical properties of MoS_2_ nanoflakes (NFs), MoS_2_- Ni, and MoS_2_-NiCNT molecular targets were described in [35]. Those results demonstrated that the bandgap of MoS_2_ NFs is increased and decreased with the addition of the nickel NPs and carbon nanotubes, respectively. The bandgap variation results from the formation of smaller-sized flakes, perturbation of electronic energy of levels, and band edge shift in flakes. Moreover, the X-diffraction measurements depicted the absence of diffraction peaks from added constituents in MoS_2_-Ni, and MoS_2_-NiCNT samples [35]. Thus, those results demonstrated that MoS_2_ NFs are the major constituents in MoS_2_-Ni, and MoS_2_-NiCNT samples whereas nickel NPs and carbon nanotubes influence the electronic, optical, and catalytic properties of MoS_2_ NFs. We used nickel NPs and CNT composites as the dopants of MoS_2_ material because of their ability to enhance the nonlinear optical properties and recombination cross-section of delocalized *π*-electron cloud, which results in the enhanced harmonic generation [28,30,31,36].

In HHG experiments, the fundamental pulse intensity must be beyond the barrier (above-barrier) suppression intensity (BSI) of targets to efficiently generate high-order harmonics. BSI is defined as the threshold laser intensity required to ionize the electron from the nucleus and formulated as ~3.8 × 10^9^ IP^4^/Z^2^ W/cm^2^, where Z is an atomic number of ions and IP is an ionization potential. At the focus area, the minimum employed intensities of picosecond HP and femtosecond DP were 2.1 × 10^9^ W/cm^2^ and 1.3 × 10^14^ W/cm^2^, respectively. The targets are kept away from the focus of HPs to reduce the probability of craters formation (see Materials and Methods section). The first (and second) IP of Mo and MoS_2_ are 7.09 eV (16.1 eV) and 5.28 eV, respectively. The calculated BSI for Mo, Mo+, and MoS_2_ are ~9.6 × 10^12^ W/cm^2^, ~6.4 × 10^13^ W/cm^2^, and ~3 × 10^12^ W/cm^2^, which are lower than the minimum DP intensities. Thus, the employed intensities allow for generating high-order harmonics from the laser-induced plasmas containing ions and neutrals of molybdenum-contained molecular targets.

**Figure 1:**
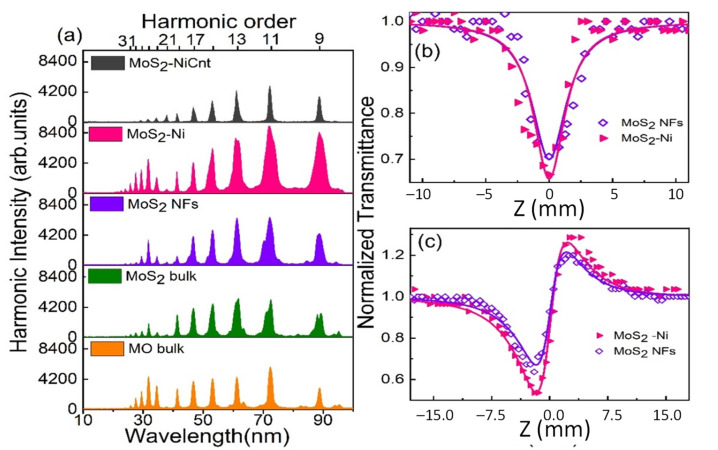
(**a**) Harmonic spectra from picosecond laser-induced plasmas of Mo-related materials. (**b**,**c**) Z-scans of MoS_2_ NFs and MoS_2_-Ni suspensions. (**b**) Open-aperture measurements. (**c**) Closed-aperture measurements. Solid curves in (**b**,**c**) show the corresponding theoretical fits.

**Figure 2:**
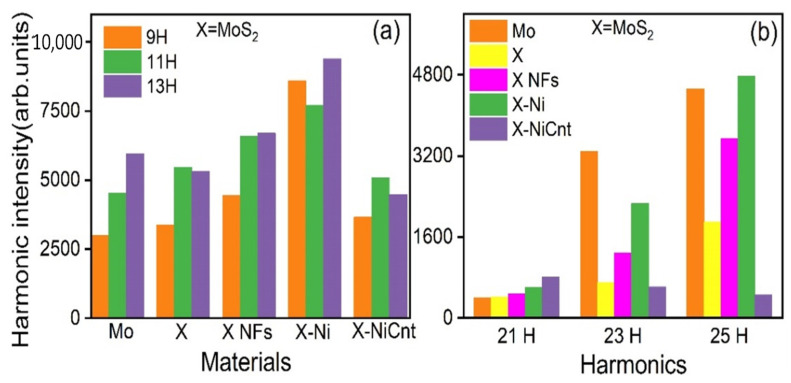
Bar diagrams of harmonic yield obtained from Mo-related materials at (**a**) 9, 11, and 13 H (**b**) 21, 23, and 25 H. X = MoS_2_.

The obtained HHG spectra from the laser-induced plasmas of studied molecular materials at 7.3 × 10^9^ W/cm^2^ (HPs) and 4.7 × 10^14^ W/cm^2^ (DPs) are illustrated in Figure 1a. For better understanding, the harmonic intensities of low-order harmonics from all ablated targets are depicted as bar diagrams in Figure 2a. The harmonic cut-offs from Mo bulk, MoS_2_ bulk, MoS_2_ NFs, MoS_2_-Ni, and MoS_2_-NiCNT plasma plumes are extended up to 31H (48.05 eV), 31H (48.05 eV), 33H (51.1 eV), 37H (57.34 eV), and 29H (44.9 eV), respectively. The obtained harmonic plateau regions of Mo bulk, MoS_2_ bulk, MoS_2_-NFs, MoS_2_-Ni, and MoS_2_-NiCNT are 13H-17H, 11H-17H, 11H-15H, 9H-13H, and 9H-13H, respectively. The observed harmonic cut-off from the atomic Mo bulk laser-induced plasma is consistent with previously reported results [18,19]. The observed cut-off energy from the plasma produced on MoS_2_ molecular bulk target is similar to the same on Mo atomic bulk target, whereas the total harmonic yield in the former case was ~10% lesser concerning the latter case. 

The total harmonic yield obtained from laser-induced plasmas of MoS_2_ NFs and MoS_2_-Ni targets is 1.4 and 2 times larger than from the plasma produced on the MoS_2_ bulk target. The total harmonic yield in that case means a summation of the peak values of all harmonics obtained from targets. The MoS_2_-Ni plasma allowed the generation of the total harmonic yield which is 1.5 times larger compared to the MoS_2_ NFs case. The main differences between nanoflakes and bulk targets are the material dimension and large surface-to-volume ratio. The MoS_2_ nanoflake’s surface texture has the potential to absorb a larger amount of heating radiation compared to the bulk material. Hence, the illumination of NFs by HPs results in a higher rate of particle ejection in plasma or, in other words, denser plasma concerning the ablation of the bulk target. The harmonic yield is proportional to the density of plasma particles. Correspondingly, the harmonic intensity induced by MoS_2_ NFs should be larger than the same by bulk MoS_2_. We did not compare the harmonic intensity by weight of different MoS_2_-containing samples. We observed that the harmonic intensity from MoS_2_ NFs was higher compared to the ablated MoS_2_ bulk at the same driving and heating pulse intensities. We attribute this result to the nanoflake’s surface texture, which has the potential to absorb a larger amount of heating radiation compared with the bulk material. 

The inclusion of particles as the dopants in MoS_2_ molecular structures enhances their performance as electronic and optoelectronic devices by modifying their low-order nonlinear optical properties [13,37,38,39,40]. The engineering of selenium doping in MoS_2_ demonstrated an enhanced second harmonic generation due to an increase in the second-order nonlinear susceptibility of this structure. The titanium, vanadium, and nickel doping enhanced the photosensitivity and electrical conductivity in 2D-MoS_2_ molecular materials. The third-order nonlinear absorption coefficient and strong nonlinear photoluminescence were exhibited from the silver NPs embedded in MoS_2_ TMDs. Moreover, Ni-doped CdS thin films and ZnS NPs produced enhanced third harmonic generation compared to the undoped species due to enhanced third-order nonlinear optical properties. Thus the knowledge of the low-order optical nonlinearities of such species becomes crucial for the determination of the potential application of those properties in different areas of optoelectronics, as well as for the determination of the relation between their low- and high-order optical nonlinearities. Below, we present the studies of the low-order nonlinear optical properties of MoS_2_ NFs and MoS_2_-Ni suspensions by applying the femtosecond Z-scan technique at 800 nm wavelength using 1.63 × 10^11^ W/cm^2^ intensity of DPs to observe any correlation with the high-order nonlinear optical properties of those doped molecular species determined during HHG experiments.

The Z-scan measurements were performed at the excitation pulse intensity 269 GW/cm^2^ in the colloidal suspensions obtained by ultrasonic dispersion of the samples in distilled water. The measured normalized transmittances of the laser pulses propagated through the samples using open-aperture and closed-aperture Z-scan arrangements are shown in Figure 1b,c, respectively. The solid curves correspond to the theoretical fits of the experimental data. The equations used for theoretical fits are taken from [41]. 

The reverse saturable absorption and the combination of two-photon absorption and nonlinear refraction were observed in the studied samples (MoS_2_ NFs and MoS_2_-Ni suspensions) in the cases of open- and closed-aperture Z-scan arrangements, respectively. The measured two-photon absorption coefficients and nonlinear refractive indices of MoS_2_ NFs (and MoS_2_-Ni) were 1.3 × 10^−11^ (1.5 × 10^−11^) cm/W and 5.2 × 10^−16^ (6.9 × 10^−16^) cm^2^/W, respectively. The Z-scans of samples showed that the nonlinear refractive indices and nonlinear absorption coefficients increased in the case of nickel dopants, which might be due to the localized surface plasmon-induced charge transfer. Interestingly, the enhanced harmonic intensity produced from MoS_2_-Ni correlates with a larger nonlinear absorption coefficient and refractive index compared to MoS_2_ NFs. The enhanced nonlinear absorption property of MoS_2_-Ni may increase the absorption of HP, which allows the formation of denser plasma. Correspondingly, stronger harmonics will be produced from the denser plasmas. Moreover, the characterization of these samples revealed that the presence of nickel NPs leads to the formation of smaller-sized NFs [35]. Earlier, the HHG experiments using small-sized NPs demonstrated a higher yield of harmonics compared with the larger-sized NPs due to enhanced surface-to-volume ratio leading to the increase of absorption [42]. In the case of larger-sized NPs, the inner atoms do not provide the harmonics due to the absorption of the XUV emission and screening of the accelerated electrons. Overall, the presence of nickel NPs affected the plasma density and MoS_2_ NFs dimensions, which cause the generation of stronger high-order harmonics.

The total harmonic yields from MoS_2_ NFs and MoS_2_-Ni plasmas were 2.5 and 1.7 times larger than those produced from MoS_2_-NiCNT plasma. One can conclude that the addition of multi-walled CNT decreases the harmonic yield. Macroscopically, the harmonic yield is influenced by the interference of the harmonics emitted from every molecular, atomic, and ionic source of the target. Whether it is constructive or destructive interference depends on the phases of generated harmonics from different sources. Typically, single-walled CNTs have a wall diameter of 2.5 nm. The multi-walled CNTs (MWCNTs) can be regarded as a group of single-walled CNTs having broader diameter distribution. As mentioned above, the diameter of our MWCNTs was in the range of 250–500 nm. Hence, the total harmonic signal from MWCNTs can be considered as a combination of the harmonics obtained from different walls of the tubes. In our experiments, the MWCNTs were not aligned in the preferred direction or orientation, which may influence the phases of the harmonics emitted from them. The differently aligned MWCNTs emitted harmonics having different phases, which modified the total harmonic yield emitted from MoS_2_-NiCNT. Notice that the aligned MWCNTs produced stronger third-harmonic emission than nonaligned MWCNTs [43]. Hence, the reduction in harmonic yield from MoS_2_-NiCnt plasmas compared with MoS_2_ NFs and MoS_2_-Ni plasmas can be attributed to the destructive interference of harmonics in the former case.

### 2.2. Resonance-Enhanced Harmonic Generation 

The resonant and suppressed harmonics were observed at around ~32 nm and ~38 nm from all Mo-contained materials except for MoS_2_-NiCNT (Figure 1a). The bar diagrams in Figure 2b represent the resonant harmonic intensity (25H) and neighboring longer-wavelength harmonics (21H and 23H). The four-step model explains the resonant-induced enhancement of single harmonic [44]. The resonant harmonic generation from Mo-contained materials is due to the influence of 4p-4d resonant transitions from the single- and double-ionized Mo [17]. The suppression of harmonics at 33–34 eV (~21H of 800 nm DP) in laser-induced plasmas of Mo materials (expect MoS_2_-NiCNT) is attributed to the contribution from the destructive interference of 4p-4d transitions with 4d orbital recombination. The behavior of resonant transitions with recombined orbitals is important in HHG because macroscopically, its yield depends on the interference of high-order harmonics generated from every ionic and atomic source. 

The resonant harmonic (25H) generated in Mo plasma was 11 and 1.7 times more intense than 21H and 27H, respectively. The resonant harmonic intensity in the case of ablated Mo is ~2 times stronger in comparison with MoS_2_ laser-induced plasmas. Moreover, the yields of resonant neighboring harmonics (23H, 27H, 29H) generated from MoS_2_ are also smaller compared with Mo plasma. Hence, our results show that the plasmas from the targets containing sulfide ions produce a less intense resonant harmonic, which indicates that those ions affect the favorable conditions of resonance-induced enhancement of a single harmonic. 

The resonant harmonic transitions are tailored by the driving beam characteristics (wavelength, intensity, etc.) and components present in HHG media. HHG in molecular plasmas containing oxides, phosphides, and selenides produces reduced or no resonant harmonics [45]. One can suggest that the detuning/shifting of the resonant transition reduces the oscillator strength (*gf*) of this transition. Hence, the decreased resonant harmonic yield in our case might be due to the modification of the 4p-4d resonant transitions influenced by sulfide ion, which may diminish the *gf* of the transition thus leading to the decay of the enhancement of single harmonic. At the same time, MoS_2_ plasma allows the generation of 21H, which is comparable with the case of Mo plasma when a similar suppressed harmonic was produced. This result shows that the influence of sulfide ions on the destructive bonding between 4p-4d resonant transitions and recombination to the 4d orbitals is negligible. For a detailed explanation of this effect, simulations need to be performed using the time-dependent density functional theory, which is out of the scope of the present article. Overall, the presence of sulfide ions in MoS_2_ plasma produced less intense resonant harmonic photons and lesser total harmonic yield once compared with the Mo plasma. 

The resonant harmonic (25H) generated in MoS_2_-Ni plasma was 2.5 and 1.4 times stronger compared with 25H produced in MoS_2_ NFs and bulk MoS_2_ plasmas, respectively. Meantime, the resonant harmonic generated in MoS_2_ bulk, MoS_2_ NFs, and MoS_2_-Ni plasmas was 4, 7, and 8 times stronger than 21H emitted from the same plasmas. The suppressed harmonic intensity (21H) produced from ablated targets corresponds to the following dependence: MoS_2_-NiCNT > MoS_2_-Ni > MoS_2_ NFs > Mo bulk ≅ MoS_2_ bulk. 

The enhanced 21H intensity from MoS_2_-Ni and MoS_2_ NFs plasmas compared with the plasmas produced on bulk Mo and MoS_2_ bulks might be due to higher plasma density leading to a larger concentration of the electrons recombined with the atoms and molecules while emitting 38 nm radiation, despite of the destructive interference of contributions from 4p-4d transitions with 4d orbitals. On the other hand, we did not observe the enhanced resonant harmonic and the suppressed 21H compared with the remaining higher-order spectrum in the case of MoS_2_-NiCNT plasmas. Moreover, the harmonic cut-off from MoS_2_-NiCNT was restricted to 27.5 nm (29H), which is the lowest cut-off compared to other Mo-contained materials used in our HHG experiments. 

The presence of misaligned MWCNTs in the target may either detune or shift the resonant harmonic transition. This modification will lead to either reduction or increase of the *gf* of this transition, which might lead to a generation of featureless harmonic distribution (i.e., without the enhancement of 25H) or demonstration of stronger enhancement of this single harmonic or other harmonics. The detuning or shifting of transition may occur due to the alternation of the refractive index of plasma at the resonant wavelength, which can lead to the modification in the phases of the interacting waves (i.e., 25H from different components of MoS_2_-NiCNT plasma). The characteristic study of MoS_2_-NiCNT [35] revealed that the addition of MWCNTs into MoS_2_-Ni leads to a change in electrical and optical properties of MoS_2_ NFs by (i) the creation of new energy levels and (ii) band edge shift in NFs due to interaction between the carbon and sulfur atoms in MoS_2_ NFs. Hence, the addition of CNTs influenced the resonant optical transition, as well as modified the selection rules between the 4p-4d orbitals presented in MoS_2_ NFs, which might lead to the generation of high-order harmonic spectra without the resonance characteristics for XUV photon at ~32 nm. 

Meanwhile, the intensity of 38 nm (21H, 33 eV) emission from laser-induced MoS_2_-NiCNT plasma was 2, 1.7, and 1.3 times stronger compared with the same from MoS_2_ bulk, MoS_2_ NFs, and Mo bulk plasmas, respectively. The enhancement of 21H from MoS_2_-NiCNT plasma compared with other materials can be considered as a result of the suppression of destructive interference of the contributions from the 4p-4d resonant transitions and its recombination with the 4d orbitals by modification in optical/electrical properties of MoS_2_ NFs due to presence of differently aligned CNTs in targets. 

Overall, the MoS_2_-Ni is a good source for emitting the intense coherent XUV photons at 32 nm and other harmonic wavelengths as compared to other targets used in our HHG experiments. 

### 2.3. Effect of Driving and Heating Pulse Intensities on HHG in Ablated Molecular Structures

**Figure 3:**
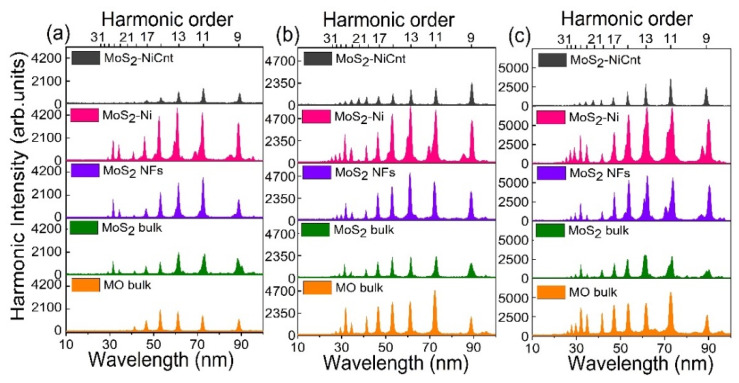
Harmonic spectra from Mo-contained plasmas at different intensities of picosecond HPs and femtosecond DPs. (**a**) 2.1 × 10^9^ W/cm^2^ and 1.3 × 10^14^ W/cm^2^. (**b**) 3.4 × 10^9^ W/cm^2^ and 2.5 × 10^14^ W/cm^2^. (**c**) 5.4 × 10^9^ W/cm^2^ and 3.4 × 10^14^ W/cm^2^.

**Figure 4:**
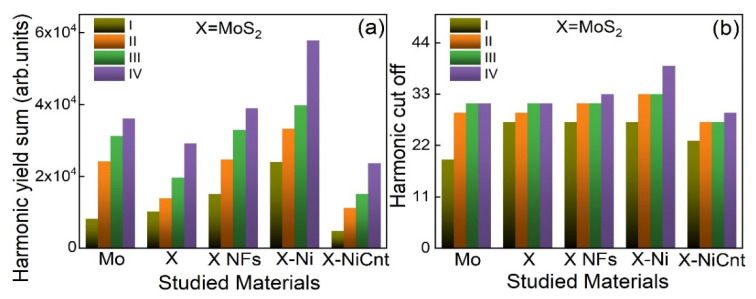
(**a**) Harmonic cut-offs and (**b**) harmonic yields at various intensities of heating and driving pulses (I: 2.1 × 10^9^ W/cm^2^ and 1.3 × 10^14^ W/cm^2^; II: 3.4 × 10^9^ W/cm^2^ and 2.5 × 10^14^ W/cm^2^; III: 5.4 × 10^9^ W/cm^2^ and 3.4 × 10^14^ W/cm^2^; IV: 7.3 × 10^9^ W/cm^2^ and 4.7 × 10^14^ W/cm^2^, X: MoS_2_).

The resonant amplification of a single harmonic due to the presence of strong ionic transitions in HHG media, as well as the total harmonic yield, can be influenced by the electric field of DPs. Hence, we studied the effect of DPs on the resonant harmonic and total harmonic yield. The dependences of high-order harmonic spectra from the plasmas comprising different Mo-contained molecular materials at various intensities of DP and HP are shown in Figure 3. For better understanding, the total harmonic yields and cut-off photon energies at various intensities of DP and HP are summarized in Figure 4a,b, respectively.

The harmonic cut-offs and intensities increased with the growth of DP and HP intensities. As per harmonic cut-off law, the maximum harmonic photon energy is defined by the relation *E*_C_ = *I_P_* + 3.17 *U_P_* where *I_P_* is ionization potential and *U_P_* is a ponderomotive potential (*U_P_* ≈ 9.33 × 10^−14^ *Iλ^2^* [W/cm^2^ × µm^2^]; here *I* is a DP intensity measured in W/cm^2^ and *λ* is the wavelength of DP measured in micrometers). One can see that cut-off energy depends on the DP wavelength and intensity. The increase in HP intensity creates higher particle density in the plasmas. Thus the enhancement in the harmonic yield and cut-off energies from Mo-contained plasmas with an increase of HP and DP intensities is attributed to the higher plasma density and ponderomotive potential, respectively. 

At 2.1 × 10^9^ W/cm^2^ (HP) and 1.3 × 10^14^ W/cm^2^ (DP) intensities, Mo bulk-contained plasma is unable to produce 32 nm (38 eV) resonant harmonic photons. Conversely, at these intensities, MoS_2_ NFs and MoS_2_-Ni produced resonant harmonic photons with their neighboring harmonics. The observed result is due to the insignificant amount of Mo ions present in plasma from Mo bulk, because of the higher ablation threshold compared to MoS_2_ NFs and MoS_2_-Ni targets. Moreover, the latter targets have higher nonlinear absorption. Therefore, at lower HP intensities, MoS_2_ NFs, and MoS_2_-Ni targets might generate larger plasma concentrations compared to the Mo bulk. Notice that, at higher DP and HP intensities, the plasma produced on the bulk Mo allowed a generation of the enhanced resonant harmonic and the neighboring high-order harmonics compared to the MoS_2_ bulk (Figure 4a). As stated earlier, it is probably due to the detuning or shifting of resonant harmonic transition or alteration of selection rules by the presence of sulfide ion in MoS_2_ plasmas, which influenced its total harmonic yield.

## 3. Discussion

MoS_2_ NFs and Nickel-doped MoS_2_ NFs allow the generation of the highest harmonic yield compared with Mo, MoS_2_ bulk, and MoS_2_-NiCnt at different employed intensities of HP and DP (see Figure 4a), which is complementary to the result discussed in Section 2.1. Overall, MoS_2_-Ni generated intense high-order harmonics at different HP and DP intensities.

As said before, the featureless and gradually decreasing pattern of harmonics from MoS_2_-NiCnt laser plasmas is due to the presence of randomly aligned CNTs influenced 4p-4d resonant transition and its interference with 4d orbitals.

Our studies showed that MoS_2_-NiCnt plasma produced the lowest harmonic yield and cut-off, while the MoS_2_-Ni plasma emitted the highest yield and cut-off energy for generating harmonics. Hence, the ablation of MoS_2_ NFs doped with nickel NPs can be employed as an effective plasma medium for HHG and demonstration of resonance enhancement at *λ* = 32 nm. Meanwhile, the stability of harmonics and the number of shots that can be obtained from the target before the harmonic intensity starts to decrease are the important parameters for efficient HHG. Considering those factors the bulk target is more beneficial compared to powder samples. Keeping these factors into consideration, the cylindrical rotating targets are used for the generation of stable high-order harmonics [46]. Hence, making thin films of MoS_2_-Ni or keeping powder samples on a motorized rotating mount can produce stable harmonics, which can find potential applications in coherent diffractive imaging and attosecond spectroscopy. 

Ni is easily oxidized, which means that the Ni NPs can be coated with NiO. The question arises how would this oxide impact the harmonic resonant? One can assume the oxidization of Ni NPs decorated on the MoS_2_ nanoflakes. The harmonic intensity depends on the plasma density, which in turn depends on the characteristics of components (ions, neutrals, composites of Ni, MoS_2_, and S, etc.). However, there will be lesser availability of NiO (ionization potential is 10.7 eV) in the plasma because the employed heating pulse intensity is higher than the barrier suppression intensity of NiO. Additionally, the nickel NPs attached to the surface of MoS_2_ nanoflakes are exposed for oxidization. Hence, the high-order harmonics generation from the MoS_2_-Ni composite is mainly from the ions and neutrals of MoS_2_, Ni, and, to some extent, Mo and S. Correspondingly, the resonance enhancement from the Mo-containing species in plasma will not be affected by the presence of the oxidized Ni. 

A similar conclusion has been reported in the case of In and In_2_O_3_ plasmas [45]. Strong enhancement of single harmonic from the indium plasma in the 62 nm range was, to some extent, suppressed once the indium oxide plasma was applied for HHG. However, the conditions of the latter plasma were changed once stronger ablation was used to the In_2_O_3_ target, leading to the enhancement of a single (13th) harmonic of the 800 nm pump propagating through such plasma. An analogous conclusion was reported in [47]. 

The MoS_2_-Ni is a semiconductor-metal (more precisely, semiconductor-oxide-metal, if Ni is oxidized) heterostructure. It has been reported that this kind of heterostructure can cause charge transfer between two (three, if Ni is oxidized) materials [48]. In laser-induced plasmas, the quantum tunneling effect enhances the resonant electron transfer cross-section in strongly coupled plasmas [49]. In our case, resonant harmonic generation is due to the influence of 4p-4d resonant transitions from the single- and double-ionized Mo component in plasma [17] rather than from MoO_y_S_x_ (before and after Ni depositions), MoS_x_, and NiS_x_ (after Ni deposition appeared in XPS spectra [48]). Wang et al. [48] claimed that the intensity of oxidization is reduced after Ni deposition. The reduction in oxidization may increase the higher harmonic intensity. 

Ni doping in MoS_2_ introduces an additional energy level [50]. The enhanced PL spectrum can be observed by tuning the incident wavelength. The decrease of PL with the growth of concentration of MoS_2_-CNT and blue shift in PL spectrum was reported in [51]. Moreover, PL spectrum of MoS_2_-BN nanotubes becomes diminished and blueshifted by adding single-walled CNT [52]. Therefore, the Ni dopants enhance the PL spectrum in MoS_2_ nanoflakes whereas the CNT quenches it. Moreover, the CNT shifts the peak of photoluminescence to the blue side.

The earlier report [35] has shown that the absorption mechanism is significantly varied by doping MoS_2_ with NiO NPs and MWCNTs, which might lead to variations in the PL intensity. They have shown that the PL yield is enhanced in molybdenum disulfide (MoS_2_)/perylene-3,4,9,10-tetracarboxylic dianhydride (MoS_2_/PTCDA) heterostructure due to reduced bang gap and reduced screening of electrons. Moreover, the shift of PL peak was observed in this heterostructure compared to the individual species due to the electron hybridization in MoS_2_ /PTCDA heterostructure. It was determined that the PL intensity is increased and the peak is blue-shifted with the increase in thickness of PTCDA organic molecule. One can expect that PL spectrum for MoS_2_-Ni becomes blueshifted due to an increase in the bandgap compared to the pristine few-layered MoS_2_ nanosheets. Recently, TMD/organic molecule heterostructures show the enhancement of PL intensity and charge carrier mobility thus allowing considering them as potential candidates for the next generation of optoelectronic devices [6].

As mentioned, HHG in the molecular plasmas containing oxides, phosphides, and selenides produced a reduced harmonic intensity. To know the exact effect of charge transfer on harmonics, high-order harmonic experiments are needed to be performed from the films as HHG medium rather than from the laser-induced plasmas. Hence, our experiments do not provide information about the charge transfer effect on the resonant harmonic generation from MoS_2_-Ni. In summary, HHG from laser-induced plasmas depends on the plasma components and their density. The plasma density is higher in MoS_2_-Ni compared to other materials since enhanced absorption leads to the production of denser plasma and stronger harmonics.

HHG spectroscopy is proven to be a useful tool for the investigation of molecular, atomic, and electronic structures of materials, as well as the analysis of the dynamics of their properties in the ultrashort time scale [53,54,55,56,57]. For spectroscopic applications, a generated bunch of high-order harmonics should be separated, which requires sophisticated XUV filters and gratings. The resonant harmonic generation diminishes the requirement for harmonic separation [58,59,60,61,62]. The demonstration of resonance-induced enhancement of harmonic in present studies can provide further insight into the amendments of harmonic yield and the developments of high-order nonlinear spectroscopy. 

The present work comprises the ablation of atomic, molecular, and complex targets for the formation of the optimal conditions for plasma formation to efficiently generate high-order harmonics. The first consideration of the comparative properties of such plasmas was reported in [63] where the high-order harmonics were analyzed from the plasma plumes prepared on the surfaces of complex targets. The studies of In–Ag targets showed that the characteristics of the high-order harmonics from the double-target plume were the same as those from the single-target plasmas. For the chromium–tellurium plasma, the enhancements of the 29th and 27th harmonics were obtained, thus indicating the appearance of the enhancement properties from both components of the double-target plasma. Those comparative studies also showed higher enhancement of a single harmonic in the case of atomic plasma (Sb) comparing to the molecular one (InSb). The additional component can only decrease the enhancement factor of the medium, due to the change in the oscillator strength and spectral distribution of the transitions involved in the resonance enhancement of the specific harmonic order. Our present studies allowed further consideration of the complex plasmas thus underlining a distinction of different mechanisms suppressing or enhancing HHG efficiency in comparison with the atomic plasmas of similar elemental content. 

## 4. Materials and Methods

**Figure 5:**
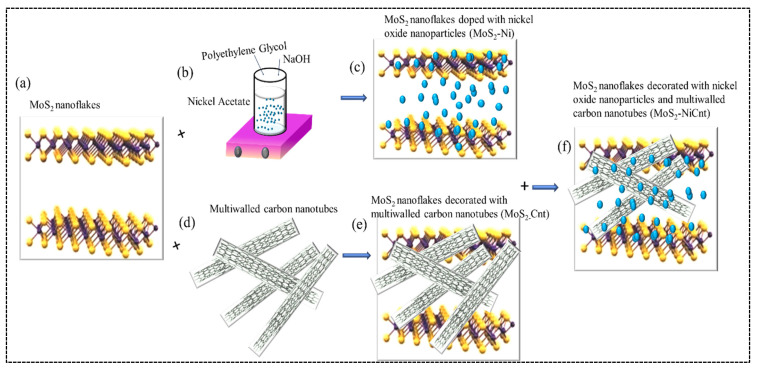
(**a**) Schematic illustration of a few-layer MoS_2_. (**b**) The synthesis scheme of Ni nanoparticles. (**c**) Schematic illustration of the doping of NiO NPs in MoS_2_ nanoflakes. (**d**) Multiwalled carbon nanotubes. (**e**) MoS_2_ nanoflakes decorated with multi-walled carbon nanotubes. (**f**) MoS_2_ nanoflakes decorated with NiO NPs and multi-walled carbon nanotubes.

Earlier, the synthesis procedure and characterization of studied samples were reported in [35]. The synthesis of MoS_2_ NFs with dopants is shown in Figure 5.

### 4.1. Synthesis of Nickel Oxide Nanoparticles 

All of the used chemicals (N-dimethylformamide (DMF), nickel acetate (Ni (CH_3_CO_2_)_2_2H_2_O), sodium hydroxide (NaOH), and sulfuric acid (H_2_SO_4_)) were purchased from Sigma-Aldrich. Without any additional processing, all of these reactants and solvents were utilized as received. We prepared the aqueous solutions using deionized water as well as ultrapure, double-distilled water.

Nickel oxide NPs were produced by chemically reducing nickel acetate with polyethylene glycol as a stabilizing agent. In this synthesis procedure, as a first step, we mixed 1 M aqueous solution of nickel acetate with polyethylene glycol (PEG) for 60 min while continuously stirring. A 1 M NaOH aqueous solution was filled into a burette tube in the vertical column and dispensed drop by drop into the nickel acetate/PEG mixture while continuously stirring. The resulting solution was then centrifuged at 5000 rpm for 10 min with 200 mL of deionized water before being stored in a glass vial for later use. The average sizes of the NiO NPs embedded in MoS_2_ were in the range of 20–50 nm depending on the conditions of synthesis. These measurements were carried out during the SEM and TEM analysis of similar samples reported in Ref. [35]. 

### 4.2. Synthesis of Multiwalled Carbon Nanotubes 

The multi-walled CNTs were synthesized through the chemical vapor deposition (CVD) technique on the Al-Cu-Fe surface. Ethylene (C_2_H_4_) was used as a carbon source in the CVD approach used to create the MWCNT on the Al-Cu-Fe substrate. The CVD chamber was evacuated and heated in an atmosphere of argon and hydrogen (Ar:H_2_ = 10:1) at a pressure of about 250 mbar. The MWCNTs were developed at 1072 K for around 20 min. Then the furnace was turned off and allowed to cool down to room temperature under the argon atmosphere. The black deposition inside the quartz tube was taken out and thoroughly washed with HCl:HNO_3_ (1:3 ratio) for 10 min followed by washing with distilled water for 60 min. Notice that an approximately similar procedure was reported in Ref. [64]. 

### 4.3. Synthesis of Few-Layer MoS_2_ and NiO Nanoparticles-Decorated MoS_2_

Few-layer MoS_2_ nanosheet sample was created by mechanically exfoliating bulk MoS_2_ powder (99.999% purity, Sigma Aldrich) in DMF solvent using a pressurized ultrasonic reactor. In brief, 50 mg MoS_2_ powder was suspended in 500 mL DMF and exfoliated for 10 h using intense ultrasonication. 2 mL of the supernatant of NiO NPs was added to the MoS_2_/DMF solution and exfoliated for another 10 h. Finally, the prepared solutions were filtered through 0.22 µm porous filter membranes and washed several times with deionized water. The final sample was vacuum dried for 12 h at 80 °C before being stored in cleaned airtight glass containers for further characterization and application.

Earlier, Lai et al. analyzed the optical, structural, vibrational, compositional, and morphological properties of a few-layered MoS_2_, as well as the NiO NPs and MWCNTs embedded on a few-layered pristine MoS_2_ [35]. Particularly, these samples show peaks at 410.3 and 384.4 cm^−1^ in their Raman spectra, which represent the A_1g_, E^1^_2g_, and E_1g_ vibrational modes of the hexagonal structure of MoS_2_. The Raman spectra of NiO NPs- and MWCNT-decorated MoS_2_ nanoflakes have shown strong peaks at 1349.5 cm^−1^ and 1604.0 cm^−1^ corresponding to the G-band related to the C-C bonds and D-band related to the defects in CNTs, respectively. Scanning electron microscopy and transmission electron microscopy confirmed the presence of Ni NPs and MWCNTs on the MoS_2_ NFs surfaces [35]. As mentioned, the average sizes of Ni NPs were in the range of 20–50 nm. The diameters of MWCNT were in the range of 250–500 nm. These species were attached to the surface of 1–4 μm MoS_2_ NFs. We did not measure the PL spectra of NiO, CNTs, and MoS_2_ flakes. 

### 4.4. HHG and Z-Scan Methods 

**Figure 6:**
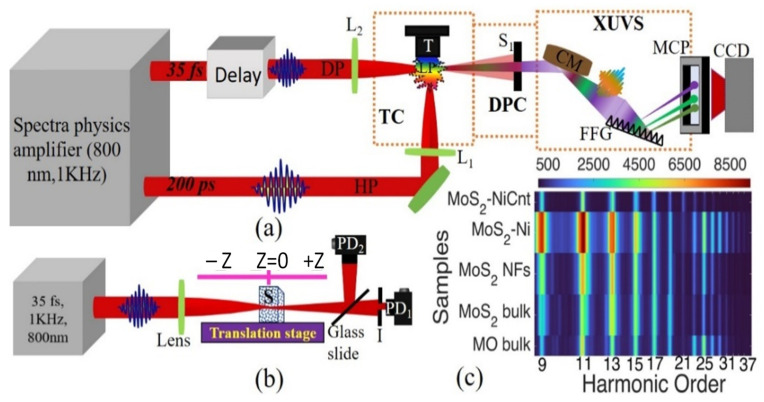
(**a**) Schematic for high-order harmonics generation in plasma. HP: heating pulses; DP: driving femtosecond pulses; L_1,2_: focusing lenses; T: target; TC: target chamber; LP: laser plasma; DPC: differential pump chamber; S_1_: slit; XUVS: extreme ultraviolet spectrometer; MCP: micro-channel plate; CCD: CCD camera; CM: cylindrical gold-coated mirror; FFG: flat-field grating; Delay: optical delay line. (**b**) Schematic for Z-Scan studies. PD_1,2_: Photodiodes, S: Samples, I: iris. (**c**) 2d color plots of harmonic spectra obtained from the molybdenum-contained materials using picosecond HP.

The experimental setup for the generation of high-order harmonics from the above-described molecular materials is presented in Figure 6a. The picosecond (800 nm, 200 ps, 1 kHz; Spitfire Ace, Spectra-Physics) pulses were used as HP for ablating the samples in the experiment. The femtosecond (800 nm, 35 fs, 1 kHz; Spitfire Ace, Spectra-Physics) laser pulses were used as DP for the generation of harmonics from the laser-induced plasmas produced on the surfaces of targets. The picosecond and femtosecond pulses were obtained from the same laser, by reflecting a small fraction of the uncompressed beam (~200 ps) before the compressor stage. The HP was focused using a spherical 300-mm focal length lens (L_1_) on the target (T) placed inside the vacuum chamber to create a laser-induced plasma. The DP was delayed by 83 ns concerning the picosecond HP and focused into the laser-induced plasmas using a spherical 500-mm focal length lens (L_2_) from the orthogonal direction to produce high-order harmonics. The DP propagated 0.2 mm above the target surface. The target and DP focusing positions are varied concerning the optical axis of DP and laser-induced plasmas, respectively, to determine the conditions for the highest yield of harmonics. 

The generated harmonics and residual fundamental radiation propagated through the differential pump chamber (DPC) and slits before entering an XUV spectrometer. The spectrometer consisted of a gold-coated cylindrical mirror (CM), a 1200 lines/mm flat field grating (FFG) with variable line spacing (Hitachi), and a microchannel plate (MCP, Hamamatsu). The images present on the phosphor screen of MCP were collected by a CCD camera (Thorlabs) coupled with a laptop to acquire information about the intensities and spectra of generated harmonics in the XUV region. The target chamber and XUV spectrometer were maintained at 10^−5^ mbar. Figure 6c illustrates the typical two-dimensional color plots of harmonics generated from different molecular plasmas. The second diffraction patterns from grating are observed on either side of the odd harmonics (i.e., between 9H-13H) in HHG spectra. It is well known that even harmonics are absent in HHG spectra from laser-induced plasmas when using the single-color DPs due to the isotropy of the HHG medium leading to the absence of the nonlinear even-order susceptibilities. The further analysis of these harmonic spectra will be explained in the following section. 

Molybdenum, molybdenum disulfide, molybdenum disulfide nanoflakes, molybdenum disulfide nanoflakes doped with nickel NPs, and molybdenum disulfide nanoflakes decorated with nickel NPs and multi-walled CNTs were used as targets of variable molecular consistency to produce laser-induced plasmas and to study the influence of the nickel and CNT on the nonlinear optical properties and generated harmonics from the Mo-contained molecular materials. 

The Mo metallic sheet of dimensions 4 mm × 4 mm and thickness 1 mm was used for ablation and atomic plasma formation. The MoS_2_ bulk molecular target was prepared in the form of a pellet having dimensions 10 mm × 10 mm with a thickness of 1 mm using a hydraulic press. The MoS_2_ NFs, MoS_2_-Ni, and MoS_2_-NiCNT targets were prepared by impinging the powdered samples on the double-sided tape (i.e., the glue of double-sided tape) attached to a glass substrate. The sizes of these targets were 4 mm × 4 mm. Notice that ablated glass substrate did not produce harmonics.

We also studied the low-order nonlinear optical properties of those materials using the Z-scan technique by illuminating the suspended molecular samples using femtosecond laser pulses. The schematic of closed- and open-aperture configurations of the Z-scan scheme is illustrated in Figure 6b. The 800 nm, 35 fs pulses were focused into a 2 mm thick quartz cell containing molecular suspensions using the convex lens having a focal length of 400 mm. The quartz cell was moved along the z-axis direction of beam propagation by controlling the movement of the translation stage through a motor controller. The reflected and transmitted parts of the propagated laser light from the glass slide were used to measure the sample’s low-order nonlinear optical properties using open- and closed-aperture schemes. In the case of an open-aperture scheme, the reflected beam was collected directly by silicon photodiode PD_2_ (PDA110A-EC, Thorlabs), whereas, in a closed-aperture configuration, only 10% of transmitted light was collected through an iris placed in front of photodiode PD_1_. 

The accuracy of Z-scan measurements depends on the characteristics of the input beam profile. Therefore, we measured the laser profile using a CCD camera. The measured laser profile was close to the Gaussian shape. The full width at half maximum of laser diameter at the focus was 38 µm. The closed and open aperture methods were used to measure the nonlinear absorption coefficients and nonlinear refractive indices of molecular samples, respectively, using 800 nm laser radiation.

## 5. Conclusions

In conclusion, we demonstrated the generation of high-order harmonics and resonance-enhanced harmonic (at ~32 nm) from the laser-induced plasmas of MoS_2_ NFs doped with nickel NPs while analyzing different Mo-containing laser-induced plasmas. The characteristics of high-order harmonics were investigated by varying the DP and HP intensities. MoS_2_ NFs doped with nickel generated the strongest coherent XUV emission than other targets. The harmonic enhancement was attributed to the increase in plasma density of MoS_2_ NFs doped with nickel. The harmonics generation in MoS_2_-NiCNT plasmas was weaker as compared with other employed targets. The randomly aligned CNTs influenced the harmonic phase leading to destructive interference, which caused a reduction in harmonic yield. Further, the resonant harmonic at ~32 nm was produced from all targets except for the MoS_2_-NiCNT. The most intense resonant harmonic was obtained in the case of MoS_2_-Ni plasma. The absence of resonant harmonic from MoS_2_-NiCNT might be due to the detuning of resonant ionic transition due to modification of the optical properties of MoS_2_ NFs by carbon nanotubes. The 38 nm harmonic intensity from MoS_2_-NiCNT plasmas was stronger compared to the one generated in other targets. This feature was attributed to the suppression of destructive interference of 4p-4d transition contributions and recombination with 4d orbitals due to the presence of carbon nanotubes in the target. The present studies elucidate an approach for using various transition metal dichalcogenides with dopants to produce intense coherent XUV radiation.

## Data Availability

Data underlying the results presented in this paper is not publicly available at this time but may be obtained from the author upon reasonable request.
